# Transcriptional Stress Induces Chromatin Relocation of the Nucleotide Excision Repair Factor XPG

**DOI:** 10.3390/ijms22126589

**Published:** 2021-06-19

**Authors:** Claudia Scalera, Giulio Ticli, Ilaria Dutto, Ornella Cazzalini, Lucia A. Stivala, Ennio Prosperi

**Affiliations:** 1 Istituto di Genetica Molecolare “Luigi Luca Cavalli-Sforza”, Consiglio Nazionale Delle Ricerche (CNR), Via Abbiategrasso 207, 27100 Pavia, Italy; C.Scalera@imb-mainz.de (C.S.); giulio.ticli@igm.cnr.it (G.T.); ilaria.dutto@gmail.com (I.D.); 2 Dipartimento di Biologia e Biotecnologie, Università di Pavia, Via Ferrata 9, 27100 Pavia, Italy; 3 Dipartimento di Medicina Molecolare, Università di Pavia, Via Ferrata 9, 27100 Pavia, Italy; ornella.cazzalini@unipv.it

**Keywords:** XPG endonuclease, chromatin association, p300/CREBBP, R loop, acetylation, genome instability

## Abstract

Endonuclease XPG participates in nucleotide excision repair (NER), in basal transcription, and in the processing of RNA/DNA hybrids (R-loops): the malfunction of these processes may cause genome instability. Here, we investigate the chromatin association of XPG during basal transcription and after transcriptional stress. The inhibition of RNA polymerase II with 5,6-dichloro-l-β-D-ribofuranosyl benzimidazole (DRB), or actinomycin D (AD), and of topoisomerase I with camptothecin (CPT) resulted in an increase in chromatin-bound XPG, with concomitant relocation by forming nuclear clusters. The cotranscriptional activators p300 and CREB-binding protein (CREBBP), endowed with lysine acetyl transferase (KAT) activity, interact with and acetylate XPG. Depletion of both KATs by RNA interference, or chemical inhibition with C646, significantly reduced XPG acetylation. However, the loss of KAT activity also resulted in increased chromatin association and the relocation of XPG, indicating that these processes were induced by transcriptional stress and not by reduced acetylation. Transcription inhibitors, including C646, triggered the R-loop formation and phosphorylation of histone H2AX (γ-H2AX). Proximity ligation assay (PLA) showed that XPG colocalized with R-loops, indicating the recruitment of the protein to these structures. These results suggest that transcriptional stress-induced XPG relocation may represent recruitment to sites of R-loop processing.

## 1. Introduction

Endonuclease XPG, belongs to the flap endonuclease (FEN) family of enzymes that cleave DNA at structure-specific sites [1,2]. For example, UV-induced DNA lesions significantly distort the double helix, generating a structure that, following DNA opening by DNA helicases, is cleaved by XPG at the 3′ end, and by XPF at the 5′ end during the nucleotide excision repair (NER) process [3,4,5]. DNA incision activity by XPG is missing in a group of patients with xeroderma pigmentosum (XP) with mutations in the ERCC5 gene coding for XPG protein [6,7]. This inactivation leads to a cancer-prone phenotype, which is also accompanied by severe deficiency in development, and by neurological disorder when XPG mutations are associated with Cockayne syndrome (CS), to produce a complex disease (XPG/CS) [8,9,10].

In addition to its fundamental role in NER, XPG participates in basal transcription, being recruited to the promoters of RNA II polymerase (pol II)-dependent genes [11,12]. This process facilitates chromatin looping and DNA demethylation reactions requiring the catalytic activity of XPG for the accurate transcription of these genes [13,14], thus explaining developmental defects induced by XPG mutations [11,12]. Furthermore, it was also shown to interact with WRN helicase and to play a role in homologous recombination (HR) induced by replication stress [15,16].

During transcription, RNA–DNA hybrids (R-loops) can be physiologically generated, but they may also result from defective transcription elongation of RNA, thus providing a source of genome instability [17]. XPG together with XPF endonuclease, are involved in the processing of R-loops, which under certain conditions may result in the formation of double-strand breaks (DSBs) [18,19]. When R-loops are formed following DNA damage, their processing requires the activity of XPG with Rad52 in order to activate transcription-associated HR [20].

The binding to DNA, the association with other factors, and chromatin recruitment are important determinants for XPG activity [21,22,23,24]. In particular, the chromatin association of NER factors needs to be regulated in a timely manner during DNA repair, since excessive retention results in genotoxic stress [25,26]. In previous studies, we showed that the XPG protein was acetylated by the lysine acetyl transferases (KAT) p300 and CREBBP (CBP) after UV irradiation, and suggested that this post-translational modification could influence the chromatin association of XPG during NER [27]. In addition, XPG was shown to be degraded after damage through the interaction with the CRL4^Cdt2^ ubiquitin ligase complex [28]. However, the mechanisms regulating XPG recruitment to chromatin and turnover during the process of basal transcription have not been specifically addressed yet.

In this study, we investigate the chromatin association of XPG during basal transcription and after transcription inhibition. We show that transcriptional stress induced by inhibitors of RNA polymerase (pol) I and II or topoisomerase I promote the chromatin accumulation of XPG in concomitance with its redistribution to nuclear clustered zones. In addition, we show that the loss of p300 and CBP KAT activity mediated by RNA interference or by chemical inhibitor C646 [29] resulted in XPG accumulation and relocalization, similarly to transcription inhibitors. Chromatin accumulation and clustered redistribution occurred in concomitance with R-loop formation and histone γ-H2AX appearance. Analysis with PLA showed that XPG was in close proximity to R-loops, suggesting that relocation induced by transcriptional stress represents the recruitment of XPG to R-loops sites.

## 2. Results

### 2.1. Chromatin Association of XPG

**Figure 1:**
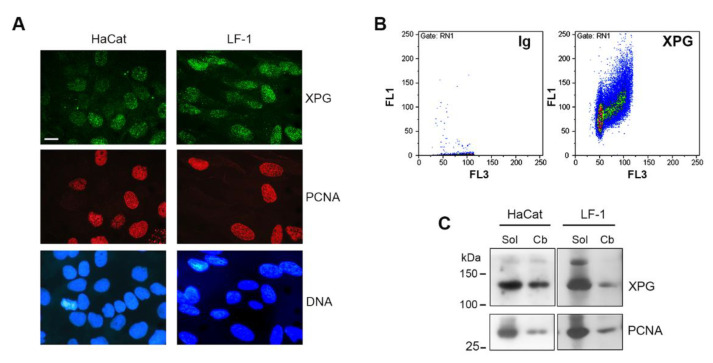
(**A**) Immunofluorescence of chromatin-bound form of XPG and PCNA proteins in HaCaT keratinocytes and in LF-1 fibroblasts. Cell grown on coverslips were subjected to in situ detergent extraction and fixed as described in Materials and Methods. XPG (green fluorescence) and PCNA (red fluorescence) shown with DNA (blue fluorescence). Scale bar = 10 μm. (**B**) Flow-cytometry dual-parameter analysis of cell-cycle distribution of chromatin-bound XPG in HaCaT cells. Immunofluorescence (FL1) was detected on cells stained with secondary antibody only (Ig), or with anti-XPG specific antibody, and counterstained with propidium iodide (PI) for DNA content (FL3). (**C**) Western blot analysis of detergent-soluble (Sol) and chromatin-bound (Cb) fractions of XPG and PCNA in HaCaT keratinocytes and LF-1 fibroblasts.

To analyze the XPG fraction that was chromatin-bound in the absence of DNA damage, we applied an in situ lysis procedure to detect chromatin-associated proteins by immunofluorescence [30]. In proliferating fibroblasts, XPG was previously shown to be present in S-phase cells [15]. Therefore, we analyzed the amount of chromatin-bound XPG under basal-growth conditions, and whether the distribution of this XPG fraction may be dependent on the cell-cycle phase. Immunofluorescence analysis was performed in proliferating HaCaT keratinocytes and in LF-1 fibroblasts. Fluorescence signals showed that high levels of the XPG protein were sometimes observed in concomitance with the presence of chromatin-bound PCNA (Figure 1A), which marks DNA replication foci under unperturbed conditions [30]. XPG association with chromatin may consistently occur in the S phase [15]. Therefore, to more quantitatively assess the cell-cycle distribution of chromatin-associated XPG, immunofluorescence was analyzed by flow cytometry. The biparametric determination of immunofluorescence vs DNA content indicated that the chromatin-bound form of XPG increased from the G1 to the S phase, reaching the highest values in the G2/M compartment (Figure 1B), resembling the distribution of a housekeeping protein. A detergent-extraction procedure, followed by DNase I digestion, was performed both on HaCaT keratinocytes and primary human LF-1 fibroblasts to obtain the unbound-soluble and chromatin-bound forms of XPG for Western blot analysis [27]. Densitometry quantification of the bands indicated that the unbound fraction, referred to as detergent-soluble protein (Sol), was predominant, while the chromatin-bound (Cb) form accounted for about 20–25% of the total XPG protein (Figure 1C). The fraction of the soluble vs the chromatin-bound form of PCNA, which is present only during the S phase in proliferating cells [30], is shown for comparison (Figure 1C).

### 2.2. Acetylation of Chromatin-Bound XPG

As mentioned above, in our previous studies, XPG protein was acetylated after UV irradiation, but some modification appeared to be present even in the absence of DNA damage in a whole cell extract of LF-1 fibroblasts [27]. Therefore, to obtain information on the chromatin-bound fraction, we analyzed the acetylation of the XPG protein, and its interaction with p300/CBP in nuclear extracts of proliferating HaCaT cells. Immunoprecipitation assays were performed with an antibody to CBP, or with antiacetyl lysine (acK) antibody, of which the specificity was previously tested [31]. Results confirmed that the XPG protein was present in both samples, indicating that, in the fraction associated with chromatin, XPG interacted with CBP, and an acetylated form could be identified (Supplementary Figure S1).

**Figure 2:**
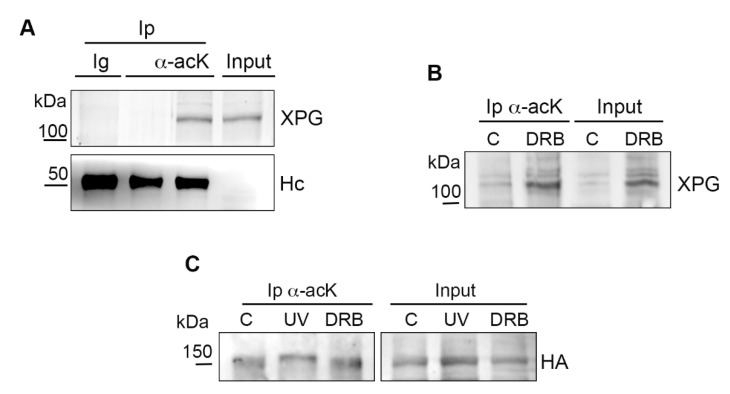
XPG acetylation is not affected by inhibition of transcription. (**A**) Specificity of immunoprecipitation (Ip) of acetylated XPG was tested in LF-1 cell extracts by incubation with purified immunoglobulin G (Ig), or with anti-acK antibody in the absence of HDAC inhibitors. Hc: Ig heavy chains. (**B**) Immunoprecipitation (Ip) of acetylated proteins with anti-acK antibody from LF-1 chromatin-bound nuclear extracts of (C) untreated or DRB-treated samples. Western blot analysis of XPG in input and Ip is shown. Input loading is 1/10 of total nuclear extracts. (**C**) Immunoprecipitation of acetylated proteins with anti acK antibody from chromatin-bound nuclear extracts of HEK293 cells expressing HA-tagged XPG. Untreated (C), UV-irradiated (10 J/m^2^), and DRB-treated samples are shown. Western blot analysis of HA-tagged XPG in the input and Ip is shown. Input loading is 1/10 of total nuclear extracts.

To investigate whether the general inhibition of transcription would affect XPG acetylation, LF-1 cells were treated for 6 h with DRB, an inhibitor of RNA pol II, and after protein extraction, an immunoprecipitation (Ip) with the anti-acK antibody was performed. The specificity of the acK antibody was tested on untreated LF-1 cells by comparing the Ip of acetylated XPG in a cell extract in the presence of the HDAC inhibitors Na butyrate and Trichostatin A (TSA) vs an extract in which these were omitted. Another control was performed by incubating a parallel aliquot of cell extract with purified IgG in place of the acK antibody (Figure 2A). In samples incubated with DRB, Western blot analysis of XPG showed that, as compared with untreated controls, the inhibitor of RNA pol II did not abolish XPG acetylation since the modified form was immunoprecipitated from nuclear extracts of both samples. In addition, analysis of the input protein showed an increase in the amount of chromatin-bound XPG in the DRB-treated sample (Figure 2B). To further validate this result, HEK cells expressing HA-tagged XPG protein [12] were similarly treated with DRB or exposed to UV radiation, and nuclear extracts were immunoprecipitated with anti-acK antibody, as above. Results showed that HA-tagged XPG was acetylated in all samples, again indicating that DRB did not inhibit XPG acetylation (Figure 2C).

**Figure 3:**
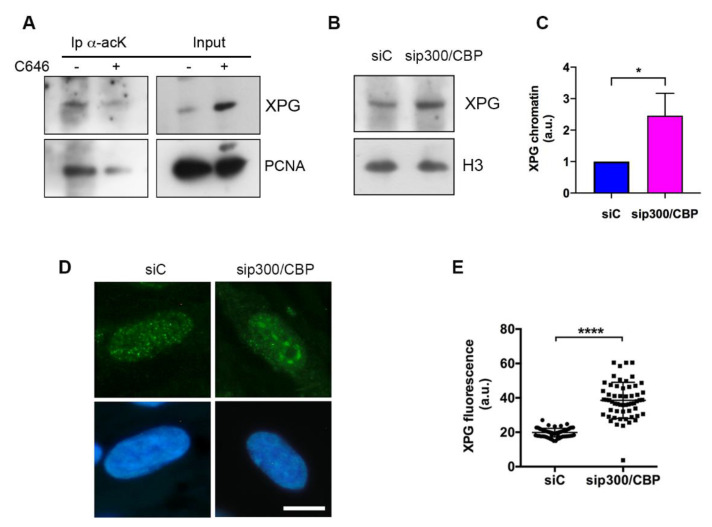
Inhibition of p300/CBP activity induces increase in and redistribution of chromatin-bound XPG. (**A**) Immunoprecipitation (Ip) of acetylated proteins from chromatin-bound nuclear extracts of (C) untreated or C646-treated LF-1 fibroblasts. Western blot analysis of XPG and PCNA in the input and Ip is shown. (**B**) Western blot analysis of XPG and histone H3 proteins in chromatin-bound nuclear extracts of LF-1 fibroblasts previously incubated with nontargeting siRNA (siC) or siRNA to p300 and CBP (sip300/CBP). (**C**) Densitometry quantification of XPG bands in the Western blots of chromatin-bound nuclear extracts of LF-1 fibroblasts previously incubated with nontargeting siRNA (siC) or siRNA to p300 and CBP (sip300/CBP). Densitometry values normalized to histone H3 content and given as fold value of the amount in siC samples. Mean values ± S.D. of three experiments are shown. (* *p* < 0.05). (**D**) Immunofluorescence staining of chromatin-bound XPG (green fluorescence) and DNA (blue fluorescence) in LF-1 fibroblasts previously incubated with nontargeting siRNA (siC) or siRNA to p300 and CBP (sip300/CBP). Scale bar = 10 μm. (**E**) Quantification of XPG fluorescence intensity in LF-1 fibroblasts prepared as in (**D**). Fluorescence was quantified by Image J as described in Materials and Methods. At least 50 cells (from two independent experiments) were analyzed. Error bars show SEM (**** *p* < 0.0001).

In order to establish the dependence of XPG chromatin association on its acetylation, immunoprecipitation with an anti-acK antibody was performed in HaCaT cells treated with C646, a specific inhibitor of p300/CBP [29]. The amount of acetylated XPG was significantly reduced in C646-treated cells (Figure 3A), as was the amount of PCNA, which is also acetylated by p300 and CBP [31]. Surprisingly, however, reduced XPG acetylation occurred in concomitance with an apparent accumulation of chromatin-bound XPG, observed in the input extract, as compared with untreated cells. To further understand whether acetylation could influence XPG chromatin association, CBP and p300 proteins were codepleted by RNA interference with siRNA in LF-1 fibroblasts, as previously described [27]. The efficiency of p300 and CBP depletion was further verified by immunofluorescence staining (Supplementary Figure S2). Western blot analysis of the chromatin-bound form of XPG in siRNA-treated LF-1 fibroblasts (Figure 3B) indicated that the depletion of both p300 and CBP induced an increase of more than two times in the level of chromatin-bound XPG after normalization to histone H3 content (Figure 3C). In addition, to investigate chromatin association at the microscopic level, we analyzed the nuclear distribution of XPG by immunofluorescence staining with a validated polyclonal antibody [32]. In LF-1 fibroblasts incubated with siRNA targeting p300 and CBP, the levels of chromatin-bound XPG showed a redistribution of XPG immunofluorescence from a fine punctuated uniform pattern, to clustered areas in a dark background (Figure 3D), with a concomitant increase in fluorescence intensity (Figure 3E). This redistribution was not observed in cells incubated with control siRNA, or targeting another protein, such as Cul4A (not shown). In order to confirm objectively the redistribution of XPG, texture analysis by using the gray level co-occurrence matrix (GLCM) was performed [33]. Results reported in Table 1 indicate that, in cells depleted of p300/CBP, the parameters of texture homogeneity (angular second moment, inversed difference moment, and correlation) were lower, while the parameter of disorganization (entropy) was higher, respectively, than the corresponding values measured in cells incubated with control siRNA oligos.

### 2.3. Inhibition of Transcription Induces XPG Accumulation and Relocation

**Figure 4:**
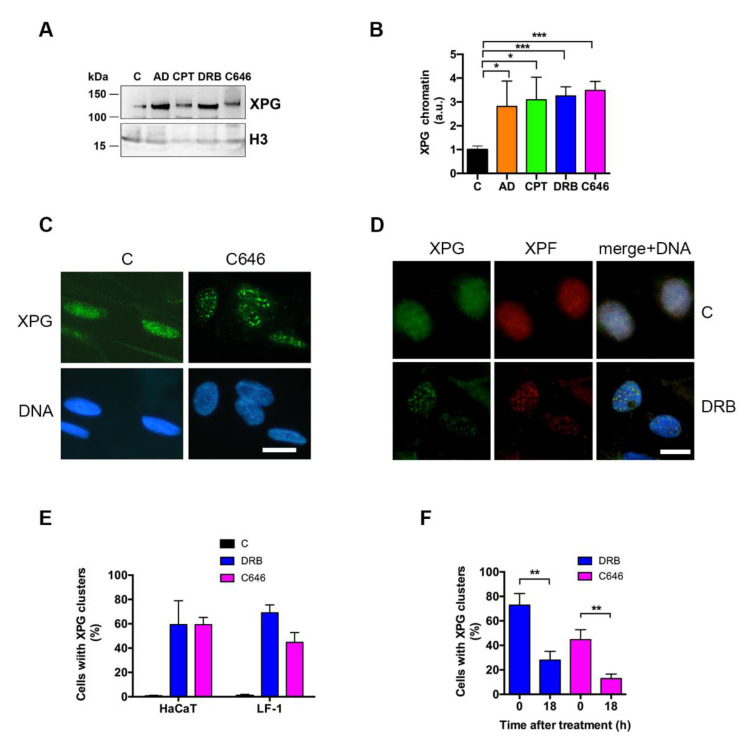
Accumulation and nuclear localization of XPG after transcription inhibition. (**A**) Western blot analysis of chromatin-bound fraction of XPG in LF-1 fibroblasts after treatment with indicated transcription inhibitors, as described in Materials and Methods; histone H3 is shown as a loading control. (**B**) Quantitative densitometric analysis of chromatin-bound fraction of XPG in LF-1 fibroblasts. Mean values ± S.D. of at least three different experiments shown. * *p* < 0.05; ** *p* < 0.01; *** *p* < 0.001. (**C)** Immunofluorescence of chromatin-bound XPG in LF-1 fibroblasts in (C) untreated and C646-treated (C646) samples. Scale bar = 10 μm. (**D**) Immunofluorescence analysis of chromatin-bound XPG (green fluorescence) and XPF (red fluorescence) in HeLa cells in untreated (C) and DRB-treated (DRB) samples. Scale bar = 10 μm. (**E**) Quantification of cells showing clustered distribution of XPG in HaCaT and in LF-1 untreated culture samples (C), or after treatment with DRB, or C646. Mean values ± S.D. of three experiments shown. (**F**) Quantification of cells showing clustered distribution of XPG in LF-1 fibroblasts at end of the treatment (0 h), and after 18 h recovery in the absence of the inhibitor. Mean values ± S.D. of three experiments shown. (** *p* < 0.01).

As both p300 and CBP are involved in basal transcription [34], the relocation of XPG could not be unequivocally attributed to a reduction in its acetylation, but could be the result of transcription impairment. In fact, C646 was also able to inhibit basal transcription as determined by BrU incorporation (Supplementary Figure S3). On the basis of these results, we sought to understand whether blocking RNA synthesis could also influence the extent and nuclear distribution of chromatin-bound XPG. To this end, LF-1 cells were treated with the RNA polymerase II (pol II) inhibitors AD or DRB, or with the topo I inhibitor CPT, in addition to C646. As compared with untreated control cells, all these compounds induced a significant increase in the amount of chromatin-bound XPG, as determined by Western blot analysis (Figure 4A). The quantification of band intensities by densitometry and normalization to histone H3 indicated that the levels of chromatin-bound XPG increased by 2–3 times, as compared with in the untreated control cells (Figure 4B). In concomitance, redistribution of XPG to nuclear clusters similar to those observed after depletion of p300/CBP was found in C646-treated cells (Figure 4C), and with all other transcription inhibitors (Table 2), including RNA pol I inhibitor BMH21 (Supplementary Figure S4). Similar clustered localization was observed for XPF, which is the other endonuclease working in concert with XPG in both NER and for R-loop processing (Figure 4D). However, the redistribution of XPG was not a general phenomenon involving nuclear proteins, since PCNA did not show similar compartmentalization (Supplementary Figure S5A).

XPG relocation was observed in both LF-1 fibroblasts and HaCaT keratinocytes (Supplementary Figure S5B), and the quantification of cells showing this pattern indicated that the phenomenon occurred independently of cell type (Figure 4E). Since transcription inhibition by DRB is known to be reversible, we also investigated whether XPG focus formation followed a similar fate. Interestingly, the removal of DRB or C646 from the medium resulted in a significant reduction in cells with XPG clusters, as seen at 18 h after the end of treatment (Figure 4F). Transcription inhibition induces the segregation to perinucleolar sites of a number of transcription-related proteins, including CDK2 [35]. Therefore, we investigated whether XPG relocation could overlap the nuclear distribution of this factor. However, after DRB treatment, the relocation of CDK2 appeared to be markedly different from that of XPG (Supplementary Figure S6).

### 2.4. XPG Relocation Occurs in Concomitance with Histone γ-H2AX and R-Loop Formation

**Figure 5:**
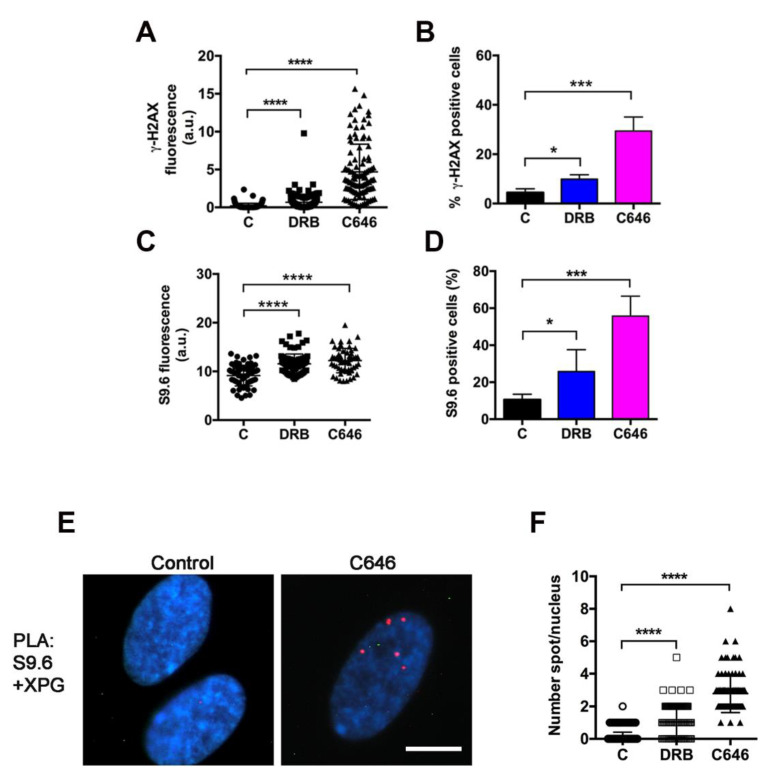
Relocalization of XPG induced by transcription inhibition occurs in concomitance with the phosphorylation of histone H2AX and R-loop formation. (**A**) Quantitative analysis of histone γ-H2AX immunofluorescence staining in LF-1 fibroblasts treated with DRB or C646. At least 50 cells (from 2–3 independent experiments) were measured. Error bars with SEM shown. (**** *p* < 0.0001). (**B**) Quantitative analysis of positivity to immunofluorescence staining of histone γ-H2AX, in LF-1 fibroblasts treated with indicated inhibitors of transcription. Mean values ± S.D. of three experiments shown. (* *p* < 0.05; *** *p* < 0.001). (**C**) Quantitative analysis of S9.6 immunofluorescence staining in LF-1 fibroblasts treated with indicated inhibitors. At least 50 cells (from 2–3 independent experiments) measured. Error bars with SEM shown. (**** *p* < 0.0001). (**D**) Quantitative analysis of positivity to immunofluorescence staining of RNA/DNA hybrids with S9.6 antibody, in LF-1 fibroblasts treated with indicated inhibitors. Mean values ± S.D. of three experiments shown. (* *p* < 0.05; *** *p*<0.001). (**E**) PLA assay performed in LF-1 fibroblasts after treatment with C646. Red spots represent the positivity of the reaction. Scale bar: 10 μm. (**F**) Quantitative analysis of the number of PLA spots/nucleus in (C) untreated, DRB-, or C646-treated LF-1 fibroblasts. Scatter plot shows pooled data from two independent experiments. Number of spots in at least 50 cells/experiment was counted. (**** *p* < 0.0001).

The inhibition of transcription by AD or CPT induces DNA damage and the phosphorylation of histone H2AX (γ-H2AX) [36,37]. Therefore, to understand whether XPG relocation was related to a similar DNA damage response, we analyzed the presence of γ-H2AX positive cells in LF-1 fibroblasts treated with DRB or C646. The quantification of immunofluorescence and the percentage of positive cells suggested that C646 was able to trigger the appearance of γ-H2AX better than DRB could, whose signal was, however, significantly higher when compared with that of untreated control cells (Figure 5A,B and Supplementary S7). Next, we investigated whether this response was induced by the formation of R-loops, since XPG is involved in the processing of such structures [18]. Immunostaining with antibody S9.6 was performed in cells treated with DRB or with C646. Results indicated that both drugs induced the appearance of cells positive to S9.6 immunofluorescence staining (Figure 5C,D and Supplementary Figure S7). To understand whether the relocation of XPG occurred in dependence of R-loop formation, their colocalization by double immunofluorescence staining could not be performed due to differences in the optimal fixation procedure for each antigen. Therefore, we analyzed the proximity of XPG to R-loops using PLA, which provides better resolution than that of confocal microscopy. Results indicated that XPG colocalized with R-loops with a higher frequency after C646 than that after DRB treatment (Figure 5E,F).

## 3. Discussion

In this study, we investigated the chromatin association of XPG during normal cell growth and after the inhibition of transcription, revealing a novel feature of XPG nuclear localization. Our results showed that this protein is accumulated at nuclear clusters when transcription is impaired both by RNA pol II and topo I inhibitors. However, this accumulation was also observed after the inhibition of p300 and CBP either with C646 or by siRNA-mediated depletion of both KAT enzymes whose activity is involved in XPG acetylation [27]. Since p300 and CBP play enzymatic and nonenzymatic functions during transcription [34], our results suggest that XPG was accumulated due to transcription inhibition, and not (at least not only) because of reduced acetylation. XPG remained acetylated after treatment with DRB, thus suggesting that the redistribution of XPG as nuclear clusters was not specifically related to XPG acetylation.

Some nuclear proteins involved in transcription may undergo redistribution by forming nucleolar caps after treatment with AD or DRB [35]. Although a similarity was observed with XPG relocation, the process appears to be different, since we did not find correlation of localization between XPG and CDK2, one of the factors observed to form these caps during transcription inhibition [35]. In this experiment, we had to use an anti-XPG monoclonal antibody (instead of the polyclonal), to assess the colocalization of XPG and CDK2. Therefore, a difference in epitope binding might have influenced these results, and further analysis is required.

Our results showed that XPG accumulated in regions in which XPF was also present, suggesting that relocation was related to a function involving both endonucleases, such as the processing of R-loops [18]. In fact, both DRB and C646 induced the formation of RNA/DNA hybrids, as detected by S9.6 immunofluorescence. PLA confirmed that the inhibition of transcription induced XPG redistribution at the same sites in which R-loops were detected. The evidence that, in our experimental conditions, the formation of R-loops (and of γ-H2AX) was higher with C646 than that with DRB treatment may be explained by their different mechanisms [29,38]. DRB may have blocked RNA synthesis, thereby reducing the formation of new R-loops during the treatment period. In contrast, with C646 being an inhibitor of p300/CBP, it may have impaired RNA synthesis with an indirect mechanism while still allowing for the formation and accumulation of R-loops. XPG accumulation was induced by both drugs, suggesting that the recruitment of XPG was triggered by a mechanism sensing the inhibition of RNA synthesis rather than the R-loop structure itself. Further studies would clarify this aspect.

In conclusion, our results showed that XPG is relocated to nuclear clusters after transcriptional stress, suggesting that this new distribution reflects the processing of R-loops.

## 4. Materials and Methods

### 4.1. Cell Cultures and Treatments

LF-1 human normal embryonic fibroblasts (from J. Sedivy, Brown University, Providence, RI, USA) were grown in MEM supplemented with 10% fetal bovine serum (FBS), streptomycin (100 μg/mL)/penicillin (100 IU), and 1 mM glutamine. Human immortalized keratinocyte cell line HaCaT (IZLER, BS, Italy) was grown in DMEM high-glucose medium supplemented with 10% FBS, streptomycin (200 μg/mL)/penicillin (200 IU), and 2 mM glutamine. Human epithelial kidney 293 (HEK 293) and HeLa cells were grown in DMEM high glucose as above. HaCaT and LF-1 cells were treated with 20 µΜ C646 (Selleck Chem., Houston, TX, USA) in serum-free medium for 2 h at 37 °C. Then, the serum was added to the medium, and the treatment continued for 4 h. 5,6-Dichloro-1-β-D-ribofuranosylbenzimidazole (DRB), camptothecin (CPT), and actinomycin D (AD) were obtained from Sigma Aldrich (Milano, Italy). DRB was used at 100 μM for HaCaT cells and 50 µM for LF-1 cells in complete medium for 6 h at 37 °C. Treatments with CPT (5 μM) or AD (10 μg/mL) were also performed in complete medium for 6 h at 37 °C. RNA pol I inhibitor BMH21 [39], obtained from TargetMol (Boston, MA, USA), was used at the final concentration of 2 μM. In some experiments, cells were exposed to UV-C irradiation (10 J/m^2^) with a TUV-9 lamp (Philips) and collected 30 min later [27,31].

Small-interfering (si) RNA oligos for p300 and CBP (ON-TARGET PLUS smart pool) were obtained from Dharmacon (Horizon Discovery, Cambridge, U.K.). Depletion of p300 and CBP was performed by incubating LF-1 fibroblasts for 72 h with oligos (20 nM each) to p300 and CBP, using INTERFERin (Polyplus, Illkirch, France) as transfection reagent. Nontargeting siRNA (Dharmacon) was used as control.

### 4.2. Flow Cytometry

HaCaT cells were harvested with trypsin, transferred to 15 mL tubes, centrifuged for 5 min at 300× *g* at RT, and washed with PBS. Samples were fixed with 0.5% formaldehyde in PBS for 5 min at RT, then centrifuged and resuspended in physiological saline solution to which ethanol was added to a final 70% concentration for storing at −20 °C. After thawing, cells were washed in PBS and incubated in blocking solution (1% BSA in PBT) for 30 min. Cells were then incubated at RT for 1 h with anti-XPG polyclonal antibody (Sigma, 1:200), followed by three washes in 1% BSA in PBS and incubation for 30 min at RT with secondary Alexa 488-conjugated antirabbit antibody (1:200). At the end, cells were again washed 3 times in PBT and incubated overnight a 4 °C in a solution containing 50 µg/mL propidium iodide (PI) and 0.5 mg/mL RNase A (Sigma) in PBS. Fluorescence signals were measured using a Partec PAS II flow cytometer (Partec, Münster, Germany).

### 4.3. Western Blot and Immunoprecipitation

Protein extraction was performed with a hypotonic buffer (1 mL/10^7^ cells) containing 10 mM Tris-HCl (pH 8.0), 2.5 mM MgCl_2_, 10 mM Na β-glycerophosphate, 0.5% Igepal, 1 mM PMSF, 1 mM DTT, 1 mM Na_3_VO_4_, 10 mM Na butyrate, 200 ng/mL TSA (Sigma), protease and phosphatase inhibitor cocktails (Sigma), for 8 min in ice. The detergent-soluble fraction was taken apart, and after two washes in isotonic buffer containing 10 mM Tris-HCl (pH 8.0), 150 mM NaCl, and inhibitors as above, nuclear pellets were incubated for 20 min at 4 °C with 20 U/10^6^ cells of DNase I (Sigma) in half volume of initial lysis, in 10 mM Tris-HCl buffer (pH 8.0), 2.5 mM MgCl_2_ and 20 mM NaCl, as described [27,30]. At the end of the reaction, the total volume was mixed with loading buffer. Samples were run on NuPAGE 4–12% gels (ThermoFisher) and transferred to nitrocellulose for Western blot analysis. Membranes were blocked in 5% BSA in PBS containing 0.2% Tween 20, then incubated for 1 h in primary antibody (listed in Table S1) solution in blocking buffer. After several washes in the same buffer, and incubation with relevant HRP-conjugated secondary antibody, the reaction was developed with chemiluminescence substrates (Cyanagen, Bologna, Italy), and signals were detected with a Westar R Imager (HiTech Cyanagen, Bologna, Italy).

For immunoprecipitation, the chromatin-bound fraction was diluted in isotonic buffer containing inhibitor cocktails as above, immunoprecipitated with antiacetyl lysine (acK) mouse monoclonal antibody (clone 4G12, Millipore), or with rabbit polyclonal antibody anti-CBP (Santa Cruz Biotech., St. Cruz, CA, USA), previously coupled to protein G-magnetic beads (Dynabeads, ThermoFisher, Monza, Italy) and incubated at 4 °C,for 3 h on a rotating wheel. Immunocomplexes were isolated and washed three times in the same buffer, mixed with loading buffer, and analyzed by Western blot with anti-XPG or other relevant antibodies (Table S1). The specificity of the anti-acK antibody (31) was tested by incubating cell extracts in the absence of HDAC inhibitors Na butyrate and TSA. Another control consisted of purified mouse IgG (Sigma) in place of the anti-acK antibody.

### 4.4. Immunofluorescence

Immunofluorescence detection of chromatin-bound XPG was performed in LF-1 fibroblasts) or HaCaT cells lysed in situ with hypotonic buffer containing 10 mM Tris-HCl (pH 8.0), 3 mM MgCl_2_, 10 mM Na β-glycerophosphate, 0.1% Igepal, 0.2 mM PMSF, 0.1 mM Na_3_VO_4_, and protease inhibitors, for 8 min at 4 °C [27]. Cells washed in the same buffer without detergent and then fixed in 2% formaldehyde in PBS followed by permeabilization in 70% ethanol. After saturation with 1% BSA-0.2 Tween 20, cells were incubated for 1 h with anti-XPG polyclonal antibody (Sigma) (diluted 1:200), and anti-PCNA PC10 monoclonal antibody (Dako, Agilent, Milano, Italy) diluted 1:100. Secondary antimouse and antirabbit antibodies labeled with Alexa 594 (red fluorescence), or Dylight 488 (green fluorescence), respectively, were used. DNA was stained with Hoechst 33258.

Histone γ-H2AX detection was performed after fixation of whole cells with 2% formaldehyde in PBS followed by permeabilization in 70% ethanol, and blocking step as above. The primary antibody to γ-H2AX (clone JBW301) was diluted 1:3000, followed by the secondary antimouse antibody labeled with Alexa 488.

For R-loop detection, after treatment with DRB, C646 or CPT, cells were fixed in cold 100% methanol and stored for 15 min at –20 °C. After washing in PBS, cells were incubated with blocking solution (3% BSA, 0.1% Tween 20, 4xSSC) for 30 min at RT. Slides were washed for 5 min with PBT solution (PBS + 0.1% Tween 20) and incubated overnight at 4 °C with 50 µL of blocking solution containing the S9.6 primary antibody (1:1000). Subsequently, cells were washed three times for 10 min each with PBT and then incubated 30 min with 50 μL of solution containing 4xSSC buffer and the secondary antibody, goat antimouse Alexa-488, conjugated (1:200). To improve visualization, a subsequent step of amplification (30 min) was performed with donkey–antigoat antibody Alexa-488 conjugated (1:300). After that, slides were washed three times for 10 min each with PBT, then incubated 5 min with a solution of Hoechst 33,258 dye (0.1 µg/mL) in PBS; after two washes in PBS, slides were mounted in Mowiol. Samples were observed with an Olympus BX51 fluorescence microscope using 100x oil immersion, and pictures were captured by an Olympus C4040 digital camera. Fluorescence intensity was analyzed with Image J software (version 1.52a, NIH, Bethesda, MA, USA), using DNA images to identify nuclei, and a particle-analysis tool, as described [40]. Analysis of XPG fluorescence redistribution was performed through the gray level co-occurrence matrix (GLCM) plugin of Image J [33]. Only nuclei with at least two clearly identifiable clusters were considered for this analysis.

### 4.5. Proximity Ligation Assay

Proximity ligation assay (PLA) was performed using the Duolink^®^ PLA kit (Sigma) following the provided protocol. LF-1 cells seeded on coverslips were treated with C646 or DRB, as indicated above and then fixed with formaldehyde (2%) solution in PBS, followed by cold ethanol (70%) permeabilization. Each coverslip was incubated in a humidity chamber (1 h at 37 °C) with Duolink^®^ blocking solution, and then with 50 μL of Duolink^®^ antibody diluent containing the two primary antibodies: anti-XPG (polyclonal, St John’s Laboratory), and anti-RNA/DNA hybrid monoclonal S9.6, both diluted 1:100. After that, coverslips were washed two times (5 min each) with buffer A at RT, and incubated in a preheated humidity chamber for 1 h at 37 °C with PLUS and MINUS PLA probes diluted in Duolink^®^ antibody diluent. Then, after two further washings (5 min each), samples were incubated in a preheated humidity chamber for 30 min at 37 °C with Ligase diluted in the Duolink^®^ ligation buffer. Once the ligation step had been completed, slides were washed two times (5 min each) with buffer A at RT, and the amplification step was performed in a preheated humidity chamber for 100 min at 37 °C with polymerase diluted in the Duolink^®^ amplification buffer containing red-fluorescence-labeled nucleotides for the rolling-circle amplification. Coverslips were washed two times for 10 min each with buffer B at RT. Lastly, DNA was stained using Hoechst 33,258 (0.1 μg/mL in PBS) for 2 min. After two washes in PBS, coverslips were mounted on slides in Mowiol and observed as described above.

### 4.6. Statistical Analysis

At least three biological replicates (unless otherwise stated) were performed for each experiment. Statistical analysis was performed with Prism 6 software (GraphPad, San Diego, CA, USA) used to calculate significance with the Student *t* test (two-tailed), with *p* values < 0.05 considered to be significant.

## Figures and Tables

**Table 1:** Texture analysis of XPG immunofluorescence distribution after siRNA-mediated depletion of p300 and CBP.

		Distance		
GLCM Feature	Sample	d = 3	d = 6	d = 9
Angular second moment	siControl	0.02100 ± 0.001474	0.01375 ± 0.001066	0.01073 ± 0.0008634
	sip300/CBP	0.003390 ± 0.0009269 ****	0.002188 ± 0.0006927 ****	0.001645 ± 0.0005484 ****
Correlation	siControl	0.03145 ± 0.002600	0.02413 ± 0.001858	0.01782 ± 0.001343
	sip300/CBP	0.0004503 ± 1.790 × 10^−5^ ****	0.0004200 ± 1.565 × 10^−5^ ****	0.0003847 ± 1.319 × 10^−5^ ****
Inverse difference moment	siControl	0.5568 ± 0.01724	0.4205 ± 0.01650	0.3492 ± 0.01563
	sip300/CBP	0.2367 ± 0.01459 ****	0.1446 ± 0.01191 ****	0.1036 ± 0.009709 ****
Entropy	siControl	4.839 ± 0.1121	5.331 ± 0.1188	5.566 ± 0.1207
	sip300/CBP	7.364 ± 0.1223 ****	7.909 ± 0.1215 ****	8.171 ± 0.1185 ****

Distance in pixel (d): steps used for the GLCM analysis. **** *p* < 0.0001.

**Table 2:** Texture analysis of XPG immunofluorescence distribution after inhibition of transcription with DRB or C646.

		Distance		
GLCM Feature	Sample	d = 3	d = 6	d = 9
Angular second moment	Control	0.01605 ± 0.0009294	0.009992 ± 0.0006226	0.007657 ± 0.0004850
	DRB	0.01261 ± 0.0007157 **	0.007838 ± 0.0005743 *	0.006063 ± 0.0004919 *
	C646	0.01288 ± 0.0006540 **	0.007909 ± 0.0004422 **	0.006070 ± 0.0003523 **
Correlation	Control	0.03624 ± 0.002843	0.02972 ± 0.002082	0.02411 ± 0.001495
	DRB	0.02652 ± 0.001212 ***	0.02175 ± 0.0009079 ***	0.01731 ± 0.0006764 ****
	C646	0.01612 ± 0.0009309 ****	0.01340 ± 0.0007202 ****	0.01073 ± 0.0005686 ****
Inverse difference moment	Control	0.6188 ± 0.007871	0.4613 ± 0.008658	0.3737 ± 0.008313
	DRB	0.5411 ± 0.009005 ****	0.3875 ± 0.008952 ****	0.3106 ± 0.008126 ****
	C646	0.5361 ± 0.009026 ****	0.3875 ± 0.009044 ****	0.3115 ± 0.008228 ****
Entropy	Control	4.561 ± 0.04921	5.049 ± 0.05104	5.309 ± 0.05207
	DRB	4.953 ± 0.03865 ****	5.463 ± 0.03992 ****	5.719 ± 0.03912 ****
	C646	5.041 ± 0.04941 ****	5.541 ± 0.05167 ****	5.794 ± 0.05214 ****

Distance in pixel (d): steps used for the GLCM analysis. * *p* <0.05; ** *p* <0.01; *** *p*<0.001; **** *p* <0.0001.

## Data Availability

Data presented in this study are available upon reasonable request to the corresponding author.

## Supplementary Materials

The following are available online at https://www.mdpi.com/article/10.3390/ijms22126589/s1.
