# Supplementary material for: Transcriptional Stress Induces Chromatin Relocation of the Nucleotide Excision Repair Factor XPG
Source: Int J Mol Sci. 2021 Jun 19;22(12):6589. doi: 10.3390/ijms22126589 (PMC8235791; doi:10.3390/ijms22126589)
Supplement: Supplementary file 1 [file ijms-22-06589-s001.zip › Supplementary_Figures_rev2.pdf]

Scalera et al.

Transcriptional stress induces chromatin relocation of the nucleotide excision repair factor XPG

Supplementary Figures

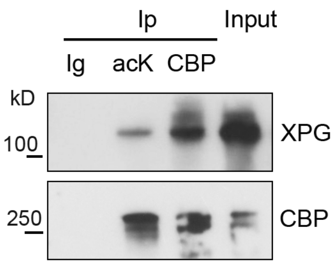

Figure S1. Immunoprecipitation (Ip) of acetylated proteins or CBP, from chromatin-bound nuclear extracts of HaCaT keratinocytes. Western blot analysis was performed with anti-XPG or CBP antibodies.

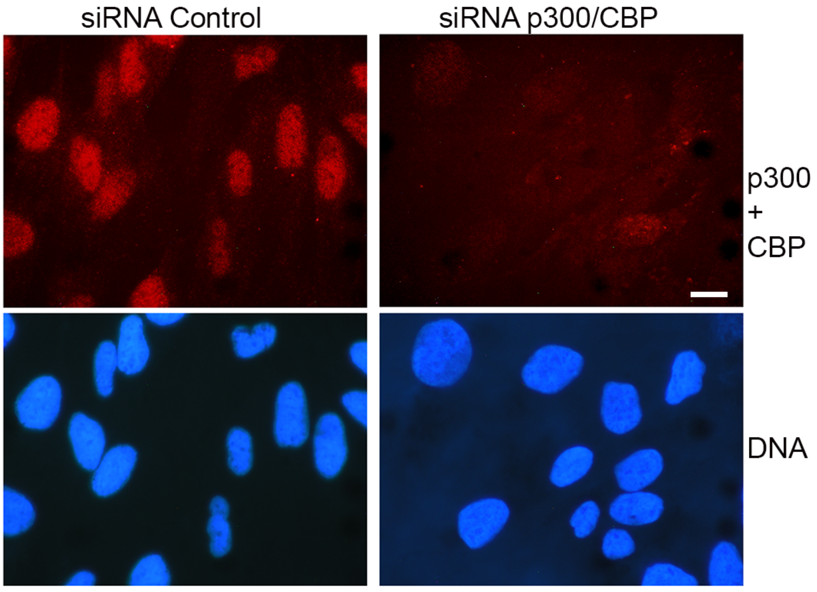

Figure S2. Depletion of p300 and CBP proteins by RNAi with specific siRNA (as described in Materials and Methods). Immunofluorescence staining (red fluorescence) of both p300 and CBP was performed with NM11 mouse monoclonal antibody recognizing both proteins. Blue fluorescence shows nuclear DNA stained with Hoechst 33258. Scale bar: 10 μm.

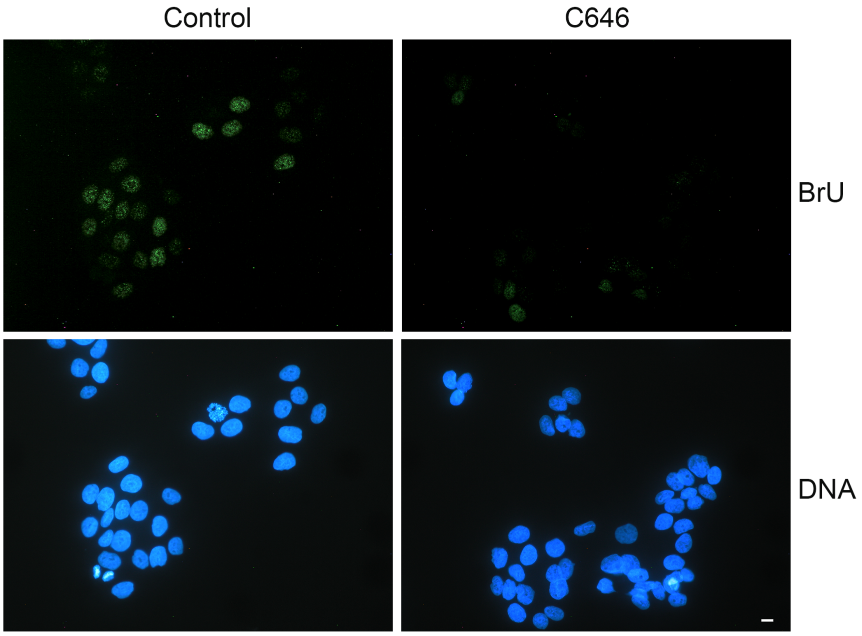

Figure S3. Determination of transcription by BrU incorporation in HaCaT keratinocytes. Cells were incubated for 6 h in the presence of 20 μM C646. During the last h, 1 mM BrU was added to the medium and then cells were fixed with 4% formaldehyde in PBS. BrU incorporation was detected with anti BrdU antibody (1:50). DNA was stained with Hoechst 33258. Scale bar: 10 μm.

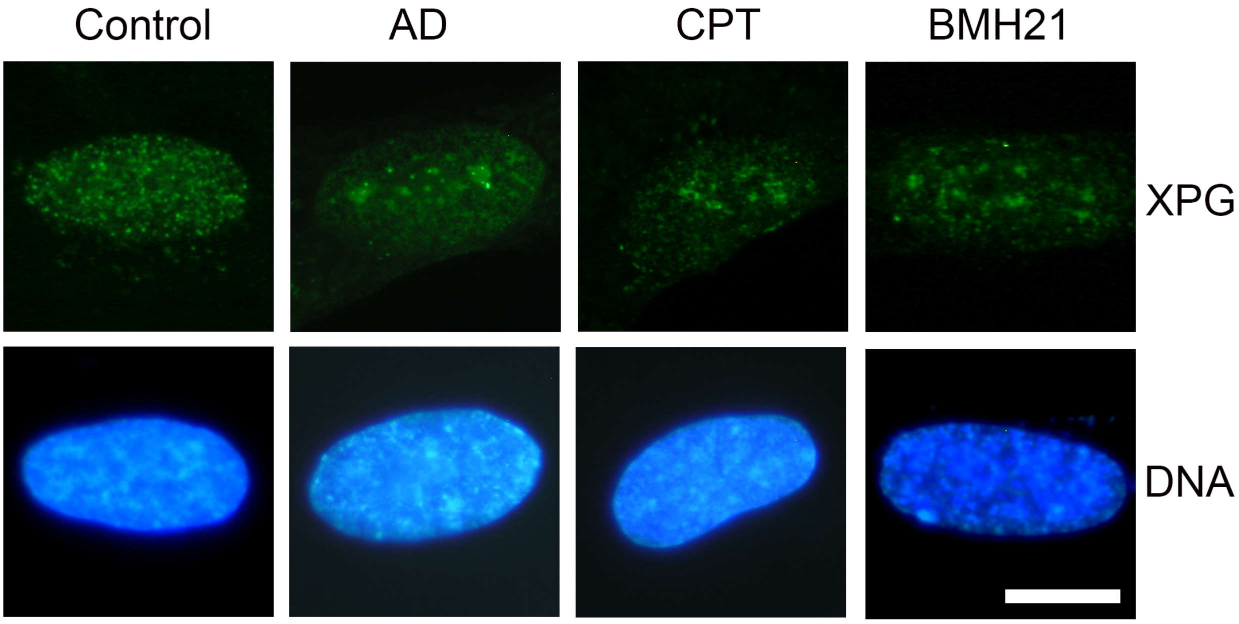

Figure S4. XPG re-localization occurs after treatment for 6 h with AD (10 μg/mL), CPT (5 μM), or BMH21 (2 μM), in LF-1 fibroblasts. After treatment, cells were lysed *in situ* and fixed (as described in Materials and methods). Immunofluorescence staining with anti-XPG antibody (Sigma), and DNA staining with Hoechst 33258, were performed as indicated. Scale bar: 10 μm.

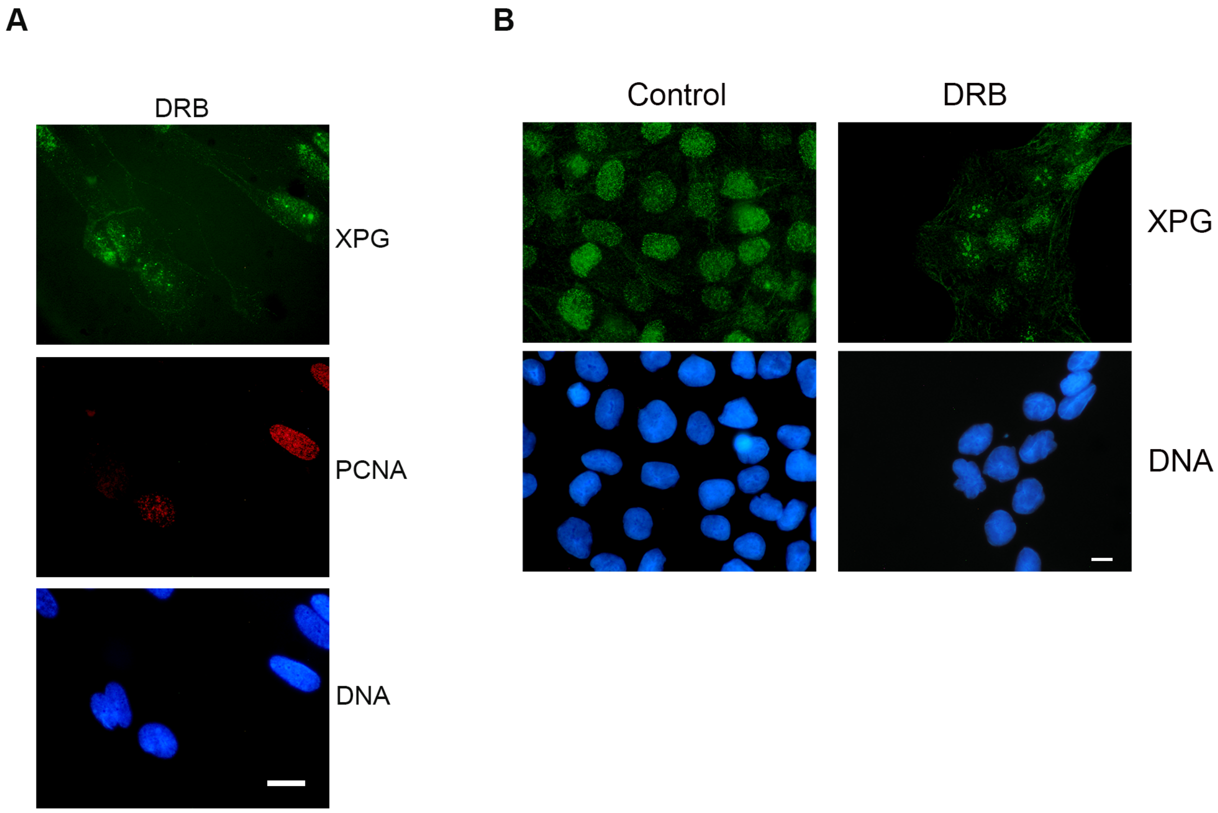

Figure S5. Specificity of XPG re-localization. (**A**) Immunofluorescence detection of XPG (green fluorescence) and PCNA (red fluorescence) in LF-1 fibroblasts treated for 6 h with 50 µM DRB. (**B**) Immunofluorescence detection of XPG (green fluorescence) in HaCaT keratinocytes treated for 6 h with 100 µM DRB. DNA was stained with Hoechst 33258 dye. Scale bar: 10 µm.

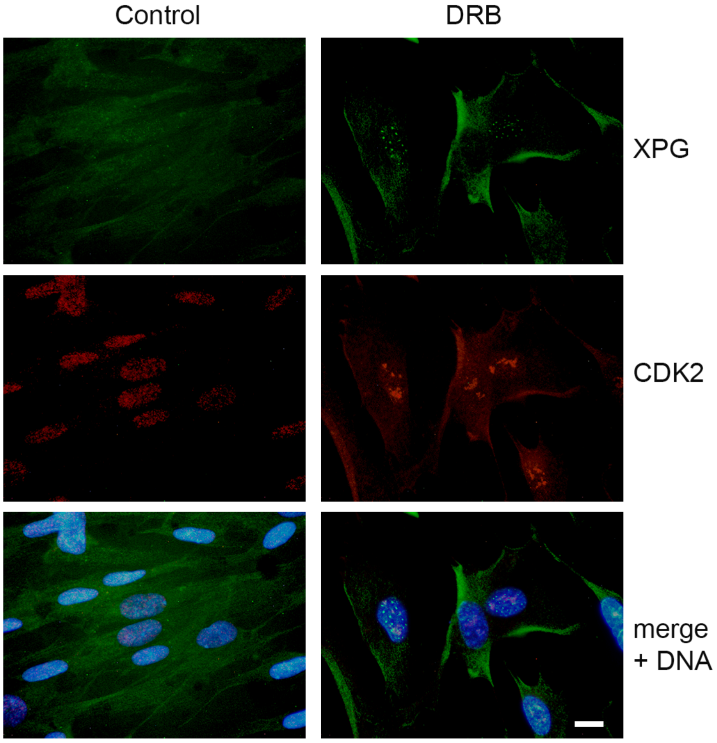

Figure S6. Immunofluorescence detection of XPG (green fluorescence) and CDK2 (red fluorescence) in untreated LF-1 fibroblasts (Control), or treated for 6 h with 50 μM DRB. Scale bar: 10 μm.

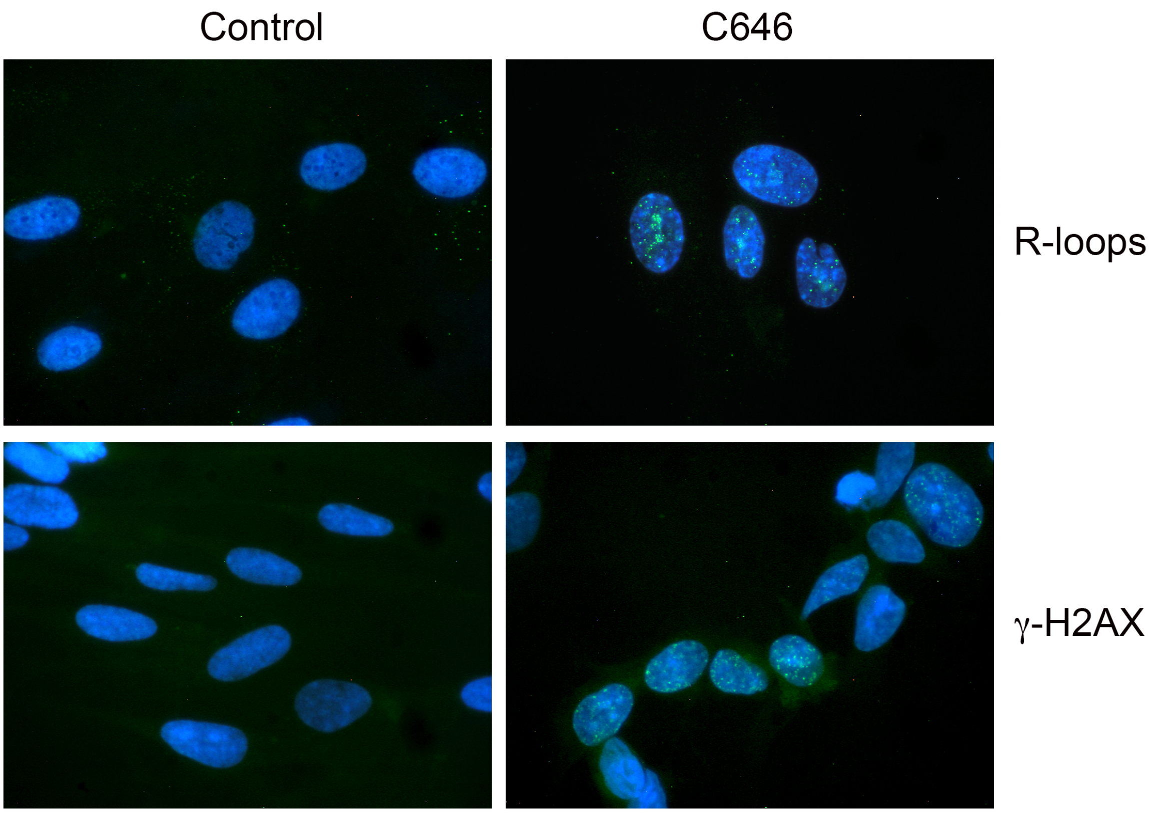

Figure S7. Immunofluorescence detection of RNA/DNA hybrids (R-loops), or of histone γ-H2AX, after treatment of LF-1 fibroblasts for 6h with C646 (20 μM). After that, untreated (Control) and treated (C646) cells were lysed *in situ* and fixed (as described in Materials and methods). Immunofluorescence staining is shown in green and merged with blue fluorescence of DNA staining with Hoechst 33258. Scale bar: 10 μm.

Table S1. List of primary antibodies used in this study

**Antigen**	**Host**	**Brand**	**Clone**
Acetyl-lysine	mouse	Millipore Merck	4G12
CBP	rabbit	Santa Cruz Biotech.	polyclonal
PCNA	mouse	Dako	PC10
XPG (C-term)	rabbit	Sigma-Aldrich	polyclonal
XPG	mouse	Santa Cruz Biotech.	8H7
XPF	mouse	Santa Cruz Biotech.	F11
γH2AX	mouse	Millipore Merck	JBW301
RNA/DNA hybrids	mouse	provided by Dr. Sabbioneda IGM-CNR	S9.6
CDK2	rabbit	Upstate (Millipore)	polyclonal
CBP/p300	mouse	Calbiochem	NM11
--	mouse	Sigma-Aldrich (I-5381)	purified IgG
